# Effectiveness of Low-Level Laser Auriculotherapy in the Treatment of Myogenic Temporomandibular Disorders and Anxiety: A Randomized, Placebo-Controlled Trial

**DOI:** 10.3390/ijerph23060697

**Published:** 2026-05-25

**Authors:** Hernán Andrés de la Barra Ortiz, Claudio Chamorro Lange, Richard Eloin Liebano

**Affiliations:** 1 Exercise and Rehabilitation Sciences Institute, School of Physical Therapy, Faculty of Rehabilitation Sciences, Universidad Andres Bello, Santiago 7591538, Chile; claudio.chamorro@unab.cl; 2 Department of Rehabilitation Sciences, University of Hartford, West Hartford, CT 06117, USA; liebano@hartford.edu

**Keywords:** laser therapy, low-level light therapy, auriculotherapy, joint disorders, temporomandibular joint dysfunction syndrome, anxiety

## Abstract

**Highlights:**

**Public health relevance—How does this work relate to a public health issue?**

- Myogenic temporomandibular disorders (TMDs) are prevalent conditions frequently accompanied by anxiety, contributing to symptom persistence and reduced quality of life.
- Non-invasive interventions addressing psycho-emotional factors are increasingly relevant in contemporary musculoskeletal rehabilitation.

**Public health significance—Why is this work of significance to public health?**

- This randomized, placebo-controlled trial shows that low-level laser auriculotherapy (LLLT-AT) may reduce anxiety when used as an adjunct to manual therapy in myogenic TMDs.
- Anxiety appears as a modifiable target not sufficiently addressed by standard physical therapy alone.

**Public health implications—What are the key implications or messages for practitioners, policy makers and/or researchers in public health?**

- LLLT-AT may represent a feasible, low-risk complementary strategy to support psycho-emotional regulation in myogenic TMDs.
- Further mechanistic and longitudinal studies are required before widespread clinical implementation.

**Abstract:**

Low-level laser auriculotherapy (LLLT-AT) is a non-invasive intervention increasingly explored for the management of musculoskeletal pain and psycho-emotional symptoms, which frequently coexist in myogenic temporomandibular disorders (TMDs). This randomized, assessor-blinded, placebo-controlled clinical trial evaluated the effectiveness of LLLT-AT as an adjunct to manual therapy in improving pressure pain threshold (PPT) and anxiety in individuals with myogenic TMDs. Forty-four participants with myogenic TMDs and clinically relevant anxiety were randomly allocated to an experimental group receiving LLLT-AT combined with a standardized myofascial release protocol (*n* =21) or to a control group receiving sham LLLT-AT with the same manual therapy (*n* =23). Interventions were delivered twice weekly for three weeks. Primary outcomes were PPT, assessed by pressure algometry, and anxiety, measured using the Generalized Anxiety Disorder–7 (GAD-7) scale. Secondary outcomes included maximum mouth opening range of motion (MMOROM) and mandibular functional limitation assessed by the Jaw Functional Limitation Scale–8 (JFLS-8). Outcomes were evaluated at baseline, post-intervention, and at a four-week follow-up. Both groups demonstrated significant within-group improvements in PPT, MMOROM, and JFLS-8 over time (*p* < 0.05), with no significant between-group differences (*p* > 0.05). Between-group analyses showed greater reductions in anxiety in the LLLT-AT group at post-intervention (*p* = 0.02; Hedges’ g = −1.35) and follow-up (*p* = 0.02; Hedges’ g = −1.68). LLLT-AT did not confer additional mechanical or functional benefits but was associated with greater reductions in anxiety when used as an adjunct to manual therapy.

## 1. Introduction

Temporomandibular disorders (TMDs) comprise a heterogeneous group of functional and/or structural conditions affecting the temporomandibular joint (TMJ), the masticatory muscles, and associated structures [1]. Clinically, TMDs are characterized by orofacial pain, limitation of mandibular range of motion, and joint sounds such as clicking or crepitus, frequently leading to impaired mandibular function and reduced quality of life [1,2]. The global prevalence of TMDs has been estimated at approximately 34%, with marked regional variation, including higher rates reported in South America (47%), Asia (33%), and Europe (29%) [3]. Recent projections suggest that the population burden of TMD may increase in the coming decades, with estimates indicating that up to approximately 44% of individuals could experience TMDs symptoms by 2050, driven in part by population ageing and changing health profiles [4]. TMDs occur more frequently in women than in men and show comparable prevalence across different age groups [5,6]. According to the Diagnostic Criteria for Temporomandibular Disorders (DC/TMDs), these conditions are classified into muscle disorders (myalgia or myogenic), joint-related disorders, and headaches attributed to TMDs [6,7]. Myogenic TMDs represent a substantial proportion of cases, particularly in clinical populations; however, their relative frequency varies depending on the population studied, diagnostic criteria applied, and case definitions, with joint-related disorders also accounting for a considerable proportion of diagnoses [7,8].

Beyond biomechanical and structural factors, myogenic TMDs are associated with psycho-emotional components, particularly anxiety and stress [9]. Anxiety has been linked to sustained muscle tension, altered pain modulation, and parafunctional behaviors such as bruxism, which may exacerbate pain intensity and functional limitation [10,11]. Evidence indicates a higher prevalence and severity of TMDs symptoms among individuals with elevated anxiety levels. However, current biopsychosocial models support a multidirectional relationship, in which psycho-emotional factors interact with biological and mechanical contributors, influencing pain perception and the risk of symptom chronification rather than acting as independent primary causes [10,11,12]. This interaction between somatic and psycho-emotional dimensions supports the need for integrative therapeutic approaches capable of addressing both components simultaneously [13,14].

Current management of myogenic TMDs includes pharmacological and non-pharmacological strategies. Pharmacological approaches commonly involve nonsteroidal anti-inflammatory drugs, muscle relaxants, analgesics, antidepressants, and anxiolytics [14]. Non-pharmacological interventions are centered on conventional physical therapy, including manual therapy, therapeutic exercise, thermal agents, and electrotherapy modalities such as transcutaneous electrical nerve stimulation and low-level laser therapy [15,16]. Complementary approaches, including acupuncture and biofeedback, have also been explored for their potential to modulate muscular activity and psycho-emotional responses, thereby improving mandibular function and symptom control [16,17].

Auriculotherapy has emerged as a complementary intervention for musculoskeletal pain and anxiety, including in TMDs populations [18,19]. This technique has been proposed to involve the stimulation of specific points on the auricle, which may function as a microsystem potentially interacting with the central nervous system through a complex innervation involving the vagus, auriculotemporal, greater auricular, and lesser occipital nerves [18,19,20]. Through these neural pathways, auricular stimulation may influence autonomic and limbic structures involved in pain processing and emotional regulation, although the underlying neurophysiological mechanisms remain incompletely understood and are still under investigation [18,21].

Several auriculotherapy modalities have been described, including needle-based acupuncture, transcutaneous electrical nerve stimulation, and percutaneous electrical nerve stimulation [17,18]. More recently, low-level laser therapy (photobiomodulation therapy) has been introduced as a non-invasive and painless alternative for auricular point stimulation, referred to as low-level laser auriculotherapy (LLLT-AT). Low-level laser therapy uses red or near-infrared light with power outputs below 0.5 W and exerts its primary effects through photobiomodulation mechanisms, including mitochondrial stimulation via cytochrome c oxidase activation and increased adenosine triphosphate production [22,23]. These mechanisms have been primarily described in experimental and preclinical studies and have been proposed to modulate inflammatory mediators, neural excitability, and endogenous opioid activity; however, their direct translation to clinical effects in myogenic TMDs remains uncertain. Clinical outcomes are heterogeneous and appear to depend on treatment parameters such as dose, wavelength, and exposure time [22,23,24,25].

LLLT-AT has gained increasing clinical interest due to its safety profile, ease of application, and potential to simultaneously influence nociceptive and psycho-emotional components of musculoskeletal pain [21,26,27]. However, despite its growing use, evidence supporting its effectiveness in myogenic TMDs remains limited, and the extent to which its effects exceed contextual or placebo-related responses remains a matter of debate [20,26,28]. In particular, randomized, placebo-controlled trials evaluating LLLT-AT as an adjunct to conventional physical therapy, with simultaneous assessment of pain-related and anxiety-related outcomes, are scarce.

Despite advances in the management of TMDs, there remains a need to explore therapeutic interventions that are not only clinically effective but also non-invasive, safe, and easy to implement in routine practice. In this context, low-level laser auriculotherapy has gained increasing interest as a promising integrative approach, given its potential to simultaneously address orofacial pain and psycho-emotional symptoms such as anxiety. However, the limited availability of randomized controlled trials restricts the ability to draw robust conclusions regarding its effectiveness in myogenic TMDs. Therefore, conducting a rigorously designed randomized, placebo-controlled trial is warranted to evaluate the effects of LLLT-AT, when used as an adjunct to manual therapy, on pain-related, functional, and anxiety-related outcomes. This study aims to contribute to the development of a more solid evidence base to support the informed integration of this intervention into clinical practice.

## 2. Materials and Methods

### 2.1. Study Design

This study was conducted as a randomized, placebo-controlled clinical trial with two parallel groups [29]. Participants were randomly allocated to either an experimental group or a placebo group, and both participants and outcome assessors were blinded to group allocation. Due to the nature of the intervention, the therapist delivering the treatment was not blinded to group allocation. However, standardized procedures were applied to minimize potential performance bias.

The trial was conducted in accordance with the Consolidated Standards of Reporting Trials (CONSORT) guidelines for randomized controlled trials [30].

The study was conducted at the Physical Agents Research Laboratory (KIN 005) of the Physical Therapy Program, Andrés Bello University, Santiago, Chile.

### 2.2. Ethical Approval and Trial Registration

The study was reviewed and approved by the Ethics Committee of the Eastern Metropolitan Health Service (EMHS) (approval date: 5 August 2025; approval No. 050825-2), ensuring compliance with the ethical principles established in the Declaration of Helsinki for research involving human participants [31]. Prior to participation, all individuals received detailed information regarding the study objectives, procedures, potential risks, and potential benefits, and provided written informed consent. The informed consent document explicitly stated the voluntary nature of participation, the confidentiality of personal data and study-related information, and the right of participants to withdraw from the study at any stage without consequences. All signed consent forms and participant data were securely stored by the principal investigator.

The clinical trial protocol was prospectively registered in the clinical trial registry of the National Institutes of Health (NIH) through the ClinicalTrials.gov platform NCT07125404 (15 August 2025) [32].

### 2.3. Study Population and Recruitment

The study population consisted of students, academic staff, and administrative personnel belonging to the Universidad Andrés Bello community, Campus Casona de Las Condes. Participants were recruited from a single university population, which should be considered when interpreting the findings.

Participant recruitment was carried out through formal institutional dissemination channels, including faculty notice boards, electronic posters, and institutional email communications. Recruitment materials provided a brief description of the study objectives, general eligibility criteria, and contact information for the principal investigator. Individuals who expressed interest in participating were screened for eligibility prior to enrollment, in accordance with the predefined inclusion and exclusion criteria.

### 2.4. Participants and Eligibility Criteria

Participants were eligible for inclusion if they were aged 18 years or older, of either sex, and presented with a unilateral or bilateral diagnosis of myogenic TMDs, established according to the DC/TMD criteria, using the Spanish-language version of the standardized diagnostic protocol applied by a trained clinician [7]. Specifically, participants presented muscle-related diagnoses including local myalgia, myofascial pain, or myofascial pain with referral, as defined by the DC/TMD classification. These diagnostic subtypes were not analyzed separately. The DC/TMD is a standardized diagnostic system based on operationalized clinical criteria and examination procedures, incorporating a decision-making algorithm that allows classification of TMDs into myogenic, joint-related, and headache-related conditions. The diagnosis required the presence of myalgia or myofascial pain affecting the masticatory muscles, either reported spontaneously or reproduced during clinical examination through palpation. Additionally, participants were required to have experienced at least one episode of local, radiating, or referred masticatory pain within the 30 days preceding the assessment, with a minimum duration of one hour [33]. To ensure the presence of psycho-emotional involvement, eligible participants were also required to present a score of ≥5 on the Generalized Anxiety Disorder 7-item scale (GAD-7), indicative of clinically relevant anxiety symptoms [34].

Participants were excluded if they met any of the following criteria: presence of recent musculoskeletal injury in the cervical region; skin lesions, wounds, or dermatological diseases affecting the auricular area; continuous use of medications such as steroidal or non-steroidal anti-inflammatory drugs or photosensitizing drugs (e.g., tetracyclines, fluoroquinolones, or sulfonamides); tattoos located on the auricular region or adjacent areas; history of cancer or tumor diagnosis within the previous five years; Fitzpatrick skin phototype V or VI [35]; autoimmune diseases (e.g., systemic lupus erythematosus), hepatic porphyria, or pellagra; epilepsy; or current use of an occlusal splint.

### 2.5. Sample Size Calculation

The sample size was calculated using G*Power software version 3.1.9.7 (Heinrich Heine University Düsseldorf, Düsseldorf, Germany) [36]. The calculation was based on a statistical power of 80% (1 − β) and a significance level of 0.05 (α), assuming a large effect size (Cohen’s d ≈ 1.0). This effect size was derived from the study by Sancakli et al. [37], which reported significant improvements in pressure pain threshold (PPT) of the masticatory muscles in patients with myogenic TMDs following four weeks of low-level laser therapy, and may represent an optimistic estimate when extrapolated to auriculotherapy interventions. Accordingly, the study was powered based on PPT, while anxiety was assessed as a clinically relevant outcome but was not used for sample size estimation, as commonly reported in TMD studies [26].

Based on these assumptions, the minimum required sample size was estimated at 28 participants, with 14 individuals allocated to each group. To account for potential dropouts or missing data during the study, the sample size was increased by 15%, based on expected attrition rates in clinical trials [38]. This adjustment was implemented to help ensure that outcome data would be available for at least 85% of the initially allocated participants, consistent with commonly accepted methodological quality standards in clinical research. Consequently, a minimum sample size of 16 participants per group was established.

### 2.6. Randomization and Group Allocation

Eligible participants were randomly assigned to one of two study groups using a simple randomization procedure: an experimental group receiving LLLT-AT and a control group receiving sham LLLT-AT. To ensure a balanced distribution of male and female participants between groups, sex-stratified randomization was applied.

The randomization sequence was generated using a web-based Research Randomizer (Version 4.0; web-based software) [39] by an independent researcher not involved in participant recruitment, assessment, or intervention delivery. Participants were enrolled by the research team following eligibility screening. Group allocation was implemented using a concealed allocation procedure, in which assignments were placed in sequentially numbered, opaque, sealed envelopes prepared in advance. The envelopes were opened only after baseline assessment and prior to the initiation of the intervention to ensure allocation concealment.

### 2.7. Interventions

The experimental group received low-level laser auriculotherapy (LLLT-AT), whereas the control group received sham low-level laser auriculotherapy (sham LLLT-AT). In addition to the allocated auriculotherapy intervention, both groups received the same base treatment consisting of bilateral myofascial release of the masseter and temporalis muscles, delivered as a standardized manual therapy protocol [38]. Participants in both groups attended two treatment sessions per week, with a two-day interval between sessions, over a total intervention period of three weeks. Thus, each participant received six treatment sessions. All interventions were delivered by the same trained therapist to ensure consistency across sessions, and treatment fidelity was supported through the use of a standardized protocol with predefined procedures.

#### 2.7.1. Low-Level Laser Auriculotherapy (LLLT-AT)

**Figure 1:**
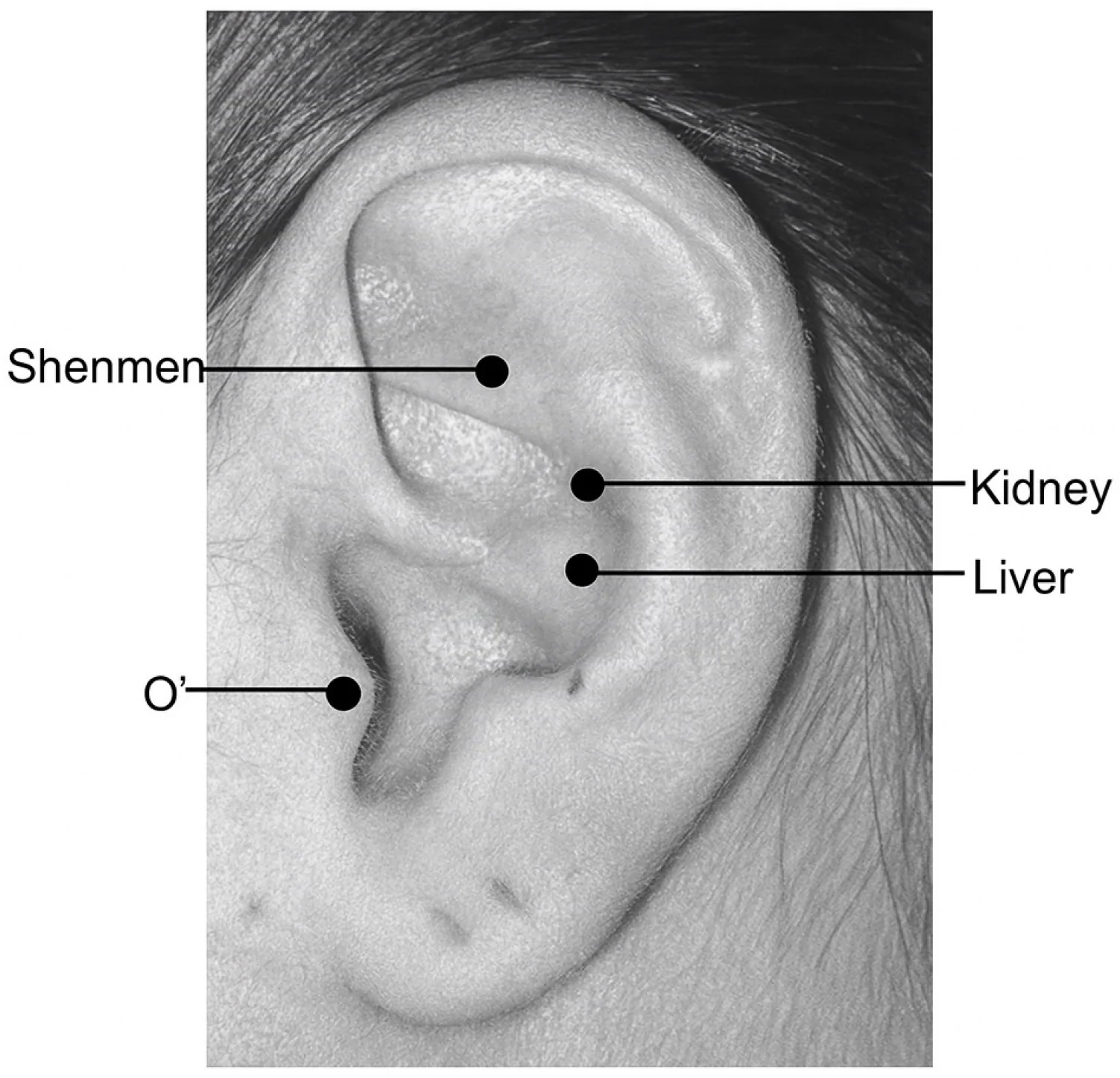
Auricular treatment points used for low-level laser auriculotherapy (LLLT-AT): Shenmen (triangular fossa), Kidney (cymba conchae), Liver (cavum conchae), and Point Zero (O′, tragus). These points are traditionally associated with analgesic effects and modulation of stress- and pain-related responses [26,40].

Participants allocated to the experimental group were treated with LLLT-AT using a low-power infrared diode laser (class IIIb) with a wavelength of 905 nm [21,22]. Laser irradiation was applied to four auricular acupuncture points: Shenmen (TF4), Kidney (AH9), Liver (AH11), and Point Zero (O′) (Figure 1). The selection of auricular points was based on both traditional auriculotherapy principles and evidence from randomized controlled trials and systematic reviews, with particular consideration of their reported clinical use in the management of anxiety and musculoskeletal pain, as well as their proposed neurophysiological relevance in autonomic and pain modulation [26,27,28,40,41]. The intervention followed the protocol described by Marques et al. [26], with an energy dose of 4 J per point.

The laser was applied with a mean output power of 71 mW for 56 s per point, resulting in a total irradiation time of 224 s and a total energy dose of 16 J per session. Auricular stimulation was applied to the ear ipsilateral to the symptomatic TMJ; in cases of bilateral pain, the auricle corresponding to the side with greater pain intensity, as reported by the participant, was selected for treatment.

During the intervention, participants were positioned in a lateral decubitus position with the treated auricle exposed. Both participants and the therapist wore protective eyewear throughout laser application. The intervention was delivered using the Combi 400L laser device (GymnaUniphy NV, Bilzen, Belgium). Additional technical specifications and laser parameters are detailed in Table 1.

#### 2.7.2. Sham Low-Level Laser Auriculotherapy (Sham LLLT-AT)

Participants allocated to the control group received sham LLLT-AT following the same procedures, positioning, treatment duration, and auricular points as the experimental group. The same laser device was used (Combi 400L laser device [GymnaUniphy NV, Bilzen, Belgium]) in both groups; however, no therapeutic laser emission was delivered during the sham application. To preserve participant blinding, all aspects of the intervention—including device handling, application time, and therapist–participant interaction—were kept identical between groups. Participants wore protective eyewear during all sessions, preventing visual identification of laser emission. This procedure was designed to control for contextual and placebo-related effects associated with auricular stimulation and therapist–participant interaction.

#### 2.7.3. Myofascial Release Therapy

Both study groups received a standardized manual therapy protocol as a baseline intervention, consisting of bilateral myofascial release techniques applied to the masticatory muscles (masseter and temporalis), following a previously described protocol [39].

- For the temporalis muscle, ischemic compression was applied to the identified trigger point for 1 min, followed by myofascial release of the trigger point for an additional 1 min. Subsequently, positional release was maintained for 30 s, followed by myofascial release of the temporalis muscle for 30 s.
- For the masseter muscle, the same procedure was applied: ischemic compression of the trigger point for 1 min, followed by myofascial release of the trigger point for 1 min, positional release for 30 s, and myofascial release of the masseter muscle for 30 s.

### 2.8. Assessment Procedures and Outcome Measures

The study evaluated both pressure pain threshold (PPT) and anxiety level (AL) as key clinical outcomes, reflecting nociceptive and psycho-emotional domains relevant to myogenic TMDs. Anxiety was included based on its established role in TMDs and its frequent assessment in previous studies, commonly using instruments such as the Beck Anxiety Inventory or the Generalized Anxiety Disorder-7 (GAD-7), often as a secondary outcome [26,41]. In the present study, the GAD-7 was selected as a validated and clinically relevant instrument to assess anxiety symptoms, while PPT was selected as the primary indicator of mechanical pain sensitivity and the variable used for sample size estimation. Secondary outcomes included changes in maximum mandibular opening range of motion (MMOROM) and mandibular functional limitation (MFL) [42,43].

All assessments were performed by an independent evaluator who was blinded to group allocation. Measurements were obtained at three predefined time points: before the initiation of treatment (T0, baseline), immediately after completion of the intervention at week three (T1, corresponding to the sixth LLLT-AT session), and at a follow-up evaluation conducted four weeks after the end of the intervention (T2).

#### 2.8.1. Pressure Pain Threshold (PPT)

**Figure 2:**
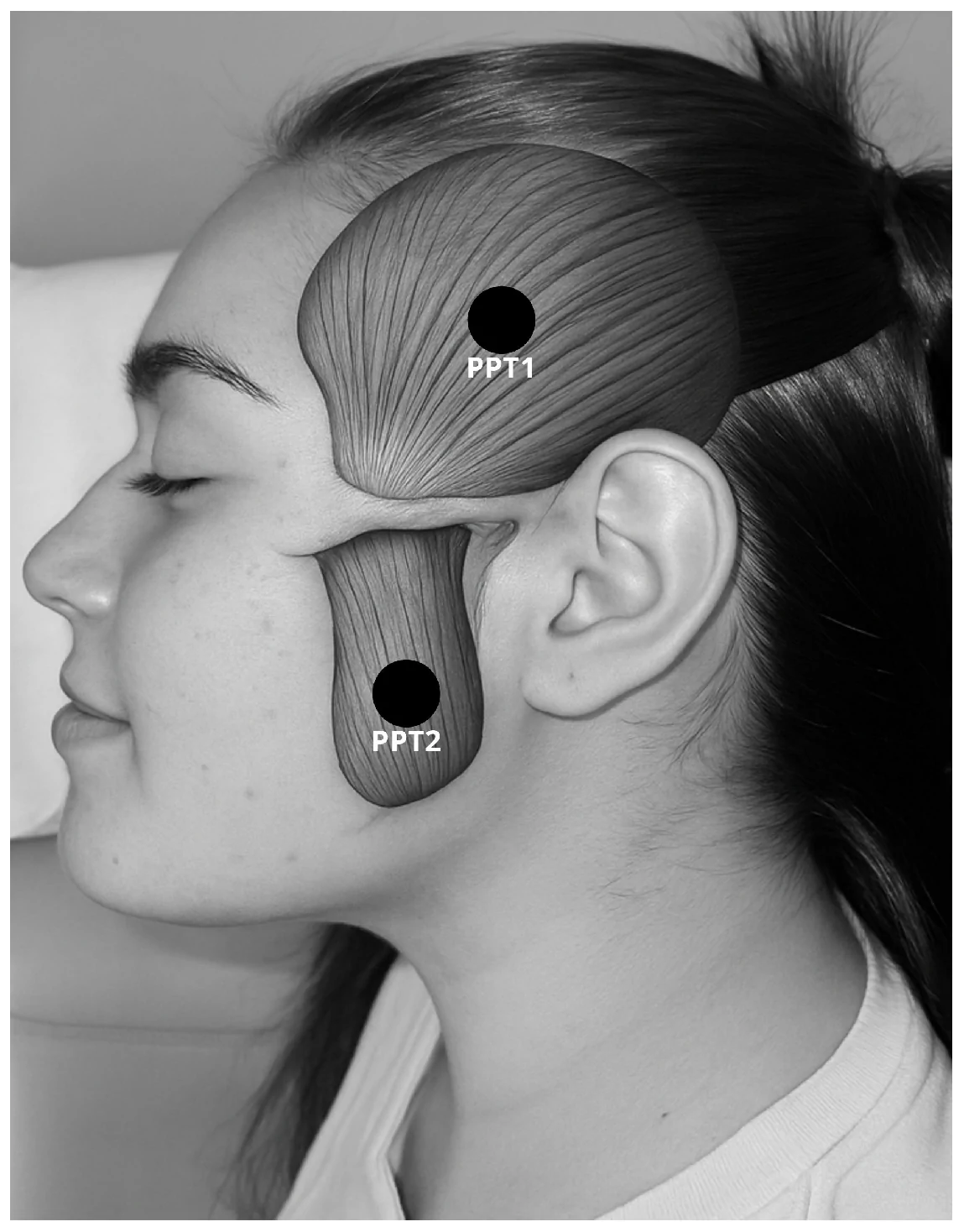
PPT assessment points in the temporalis and masseter muscles. [The authors used ChatGPT (OpenAI, GPT-5 family model) to assist in the conceptual development of the figure].

PPT was assessed using pressure algometry following a standardized protocol. Participants were positioned supine on a treatment table, and four bilateral sites corresponding to the muscle bellies of the masseter and temporalis muscles were evaluated (Figure 2) [44,45]. Each site was measured three times with a 30 s interval between measurements, and the mean value of the three trials was calculated [41,44,45,46]. The final PPT score was obtained by summing the mean values of the four assessed points and was expressed in Newtons (N). Intra-rater reliability of PPT measurements was evaluated using the intraclass correlation coefficient (ICC), calculated from repeated measurements at the midpoint of the upper trapezius muscle in 13 healthy volunteers not involved in the study, with a 48 h interval between assessments. The resulting ICC value was 0.81, indicating good reliability [44,45]. PPT was assessed using a Wagner FPX algometer (Wagner Instruments, Greenwich, CT, USA).

#### 2.8.2. Anxiety Level (AL)

AL was assessed using the Generalized Anxiety Disorder 7-item scale (GAD-7), which evaluates the frequency of anxiety-related symptoms experienced during the previous 2 weeks. Each item is scored on a Likert scale ranging from 0 (not at all) to 3 (nearly every day), and the total score is obtained by summing the responses to all seven items. The GAD-7 has been validated in Spanish and has demonstrated acceptable psychometric properties for the assessment of generalized anxiety, with a reported sensitivity of 0.83 and specificity of 0.46 [34,47].

#### 2.8.3. Maximum Mandibular Opening Range of Motion (MMOROM)

MMOROM was measured with participants in a supine position and the head in neutral alignment. Participants were instructed to perform maximal mouth opening, and the interincisal distance between the upper and lower central incisors was measured using a metric ruler and recorded in millimeters [42].

#### 2.8.4. Mandibular Functional Limitation (MFL)

Mandibular functional limitation was assessed using the 8-item Jaw Functional Limitation Scale (JFLS-8), which evaluates limitations in mandibular function across activities such as mastication, maximal mouth opening, and verbal and non-verbal communication [40,47,48]. Each item is scored on a scale from 0 (no limitation) to 10 (severe limitation). The final score for each participant was calculated as the sum of the responses across the eight items (range 0–80), with higher scores indicating greater functional limitation. Group-level values were calculated as the mean ± standard (SD) deviation of these total scores. The JFLS-8 has demonstrated high reliability (0.94–0.96), has been validated in Spanish, and shows strong correlation with the 20-item version of the instrument (r = 0.86) [48].

### 2.9. Statistical Analysis

Descriptive statistics were calculated for demographic and outcome variables, including PPT, AL, MMOROM, and MFL. Continuous variables were expressed as mean ± standard deviation. The normality of quantitative variables was assessed using the Shapiro–Wilk test [49]. A summary table of baseline demographic and clinical characteristics was generated and stratified by study group.

Baseline comparisons between groups were performed using the chi-square (χ^2^) test for categorical variables, specifically sex distribution, and independent-sample Student’s *t*-tests for continuous variables, including age, body mass index (BMI), and baseline values of PPT, AL, MMOROM, and MFL, assuming a 95% confidence level.

For inferential analysis of outcome variables, homogeneity of variances between groups was assessed using Levene’s test, and the assumption of sphericity for within-subject comparisons was evaluated using Mauchly’s test. As all variables met the assumptions of normality and homogeneity, a two-way mixed-design analysis of variance (ANOVA) with factors group (LLLT-AT vs. sham LLLT-AT) and time (T0, T1, T2) was performed. Particular attention was given to the group × time interaction effect, which was interpreted as the primary indicator of differential treatment effects between interventions. Where appropriate, post hoc pairwise comparisons were conducted using Tukey’s adjustment to control for multiple testing. In addition to *p*-values, effect sizes for interaction effects were quantified using partial eta-squared (η^2^p) to improve the interpretability of the results.

Effect sizes were calculated only for outcomes showing statistically significant between-group differences. Hedges’ g was computed at post-intervention and follow-up using group means and pooled standard deviations [50]. Effect sizes were interpreted using field-specific benchmarks for temporomandibular joint and masticatory muscle research (small ≈ 0.10, moderate ≈ 0.30, large ≥ 0.70) [51], based on empirically derived estimates from dental research [52], to enhance the clinical interpretability of the findings.

All statistical analyses were performed using IBM SPSS Statistics software (version 26). A 95% confidence level was adopted, and statistical significance was set at *p* < 0.05 for all tests. Graphical representations were generated using GraphPad Prism software (Version 10.0; GraphPad Software Inc., San Diego, CA, USA). 

## 3. Results

### 3.1. Participants and Baseline Characteristics

**Figure 3:**
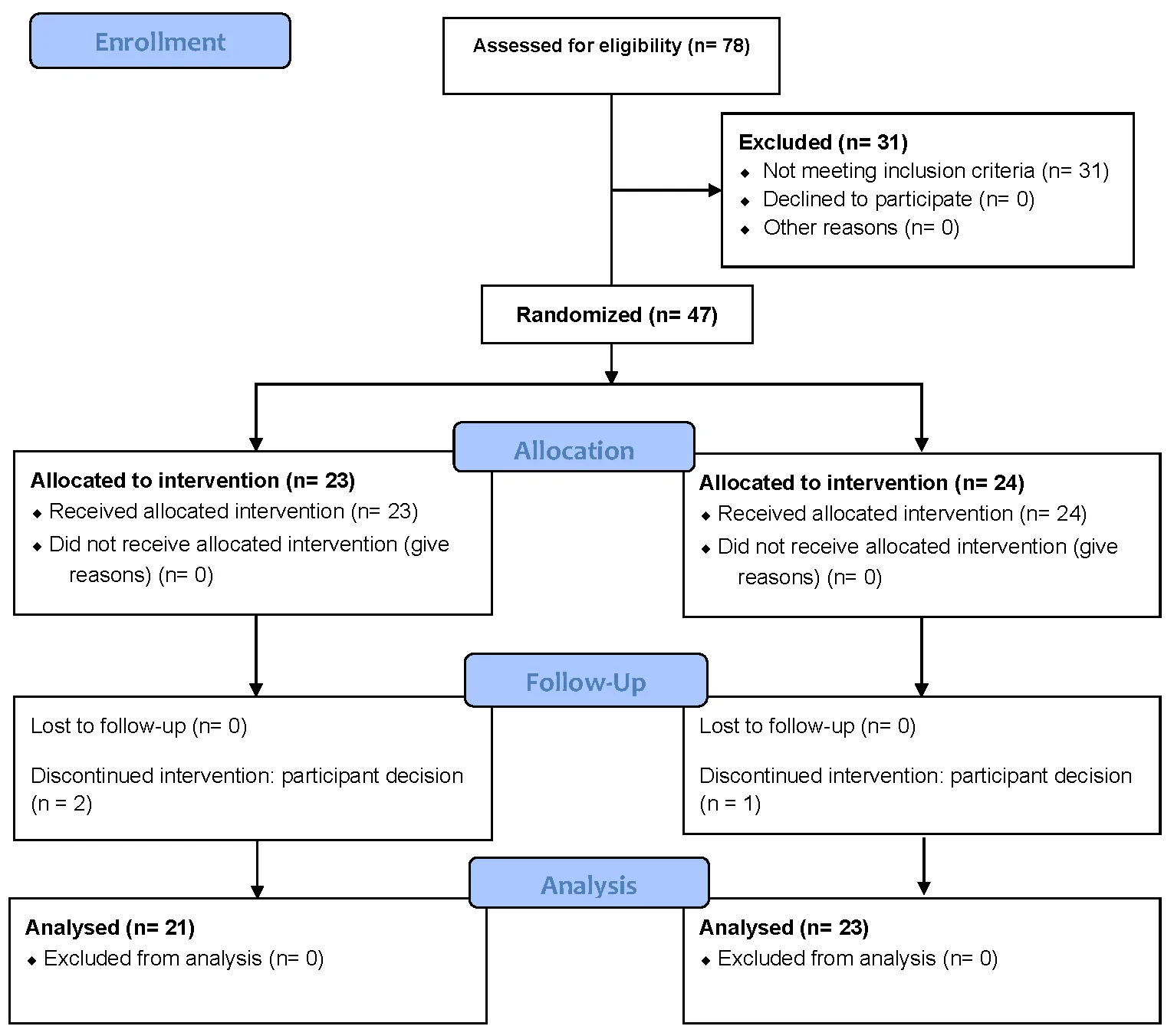
CONSORT flow diagram of participants through the RCT.

After eligibility screening, a total of 47 participants met the inclusion criteria and were enrolled in the study. The flow of participants through the different stages of the trial is presented in Figure 3 [30]. Participants were randomly allocated to the experimental group receiving LLLT-AT (*n* = 23) or to the control group receiving sham LLLT-AT (*n* = 24). During the study period, three participants withdrew, resulting in a final sample of 44 participants who completed all assessments and were included in the analysis (LLLT-AT, *n* = 21; control, *n* = 23). Baseline characteristics are reported based on the randomized sample, whereas outcome analyses were conducted using complete-case data following participant withdrawal [53].

Baseline demographic and clinical characteristics of both groups are summarized in Table 2 (based on the randomized sample). No statistically significant differences were observed between the LLLT-AT and control groups at baseline in terms of age, sex distribution, PPT at the masseter and temporalis muscles, MMOROM, MFL, or AL (all *p* > 0.05), indicating appropriate baseline comparability between groups.

### 3.2. Within-Group Outcomes in the LLLT-AT Group

In the LLLT-AT group, statistically significant improvements over time were observed across all outcome measures (Table 3). PPT increased significantly from baseline to post-intervention and remained significantly higher at follow-up in all evaluated sites of the masticatory muscles, including the right and left masseter and the right and left temporalis muscles (all *p* < 0.05).

From baseline to post-intervention (T1–T0), PPT increased at the right MAS (mean change 6.8 N; 95% CI 1.4 to 12.1), right TEM (5.4 N; 95% CI 0.2 to 10.5), left MAS (8.3 N; 95% CI 4.5 to 12.3), and left TEM muscles (8.8 N; 95% CI 3.4 to 14.3). These improvements were maintained at follow-up (T2–T0), with mean increases of 4.7 N (95% CI 1.5 to 8.4) at the right MAS, 6.7 N (95% CI 1.4 to 12.0) at the right TEM, 6.7 N (95% CI 3.7 to 9.7) at the left MAS, and 7.8 N (95% CI 3.3 to 12.3) at the left TEM, indicating a consistent and sustained enhancement of pain tolerance across the masticatory musculature.

Mandibular mobility also improved significantly following the intervention. MMOROM increased by 0.4 cm from baseline to post-intervention (95% CI 0.1 to 0.6) and by 0.7 cm from baseline to follow-up (95% CI 0.3 to 1.0; *p* < 0.01), demonstrating a maintained gain in mandibular range of motion.

In parallel, mandibular functional limitation, assessed using the JFLS-8, showed a marked and sustained improvement. Mean JFLS-8 scores decreased by 7.2 points from baseline to post-intervention (95% CI −10.8 to −3.6) and by 8.7 points from baseline to follow-up (95% CI −12.3 to −5.1; *p* < 0.01).

AL (GAD-7) also improved substantially over time, with mean reductions of 4.1 points from baseline to post-intervention (95% CI −5.9 to −2.4) and 5.0 points from baseline to follow-up (95% CI −7.0 to −3.0; *p* < 0.01), indicating a persistent reduction in anxiety following completion of the intervention.

### 3.3. Within-Group Changes in Clinical Outcomes in the Sham LLLT-AT Group

In the Sham LLLT-AT group, statistically significant changes over time were observed across several clinical outcomes (Table 4). PPT increased from baseline to post-intervention in all evaluated sites of the masticatory muscles, including the right and left masseter and temporalis muscles (all *p* < 0.05). From baseline to post-intervention (T1–T0), mean increases in PPT ranged from 4.7 N to 7.3 N, with the largest change observed in the left TEM (mean change 7.3 N; 95% CI 2.4 to 12.3).

At follow-up (T2–T0), PPT values remained significantly higher than baseline across all muscle sites, with mean increases ranging from 3.1 N to 8.4 N (all *p* ≤ 0.04). However, comparisons between post-intervention and follow-up (T2–T1) did not show statistically significant changes in PPT at any site (*p* > 0.05), indicating that the observed improvements occurred primarily during the intervention period and were subsequently maintained.

Mandibular mobility, assessed using MMOROM, showed a modest but statistically significant improvement from baseline to post-intervention (mean change 0.34 cm; 95% CI 0.1 to 0.68; *p* = 0.04), which was maintained at follow-up (mean change 0.40 cm; 95% CI 0.1 to 0.7; *p* < 0.01). No significant change was observed between post-intervention and follow-up (*p* = 0.86).

Mandibular functional limitation, assessed using the JFLS-8, decreased significantly from baseline to post-intervention (mean change −4.6 points; 95% CI −7.6 to −1.6) and from baseline to follow-up (mean change −6.3 points; 95% CI −10.5 to −2.2; both *p* < 0.01). No further significant improvement was observed between post-intervention and follow-up (*p* = 0.19).

AL also showed a significant reduction over time. Mean GAD-7 scores decreased by 2.5 points from baseline to post-intervention (95% CI −3.5 to −1.4) and by 3.2 points from baseline to follow-up (95% CI −4.2 to −2.1; both *p* < 0.01). A small but statistically significant change was observed between post-intervention and follow-up (mean change −0.7; 95% CI −1.3 to 0.1; *p* = 0.02).

### 3.4. Between-Group Differences in Clinical Outcome

Between-group analyses revealed no statistically significant differences between the LLLT-AT and Sham LLLT-AT groups for PPT at any evaluated masticatory muscle site (masseter and temporalis, bilateral), nor for MMOROM or JFLS-8, at baseline (T0), post-intervention (T1), or follow-up (T2) (all *p* > 0.05). Effect size estimates for these outcomes were small according to field-specific benchmarks for TMDs research and did not support clinically meaningful between-group differences [52] (Table 5).

**Figure 4:**
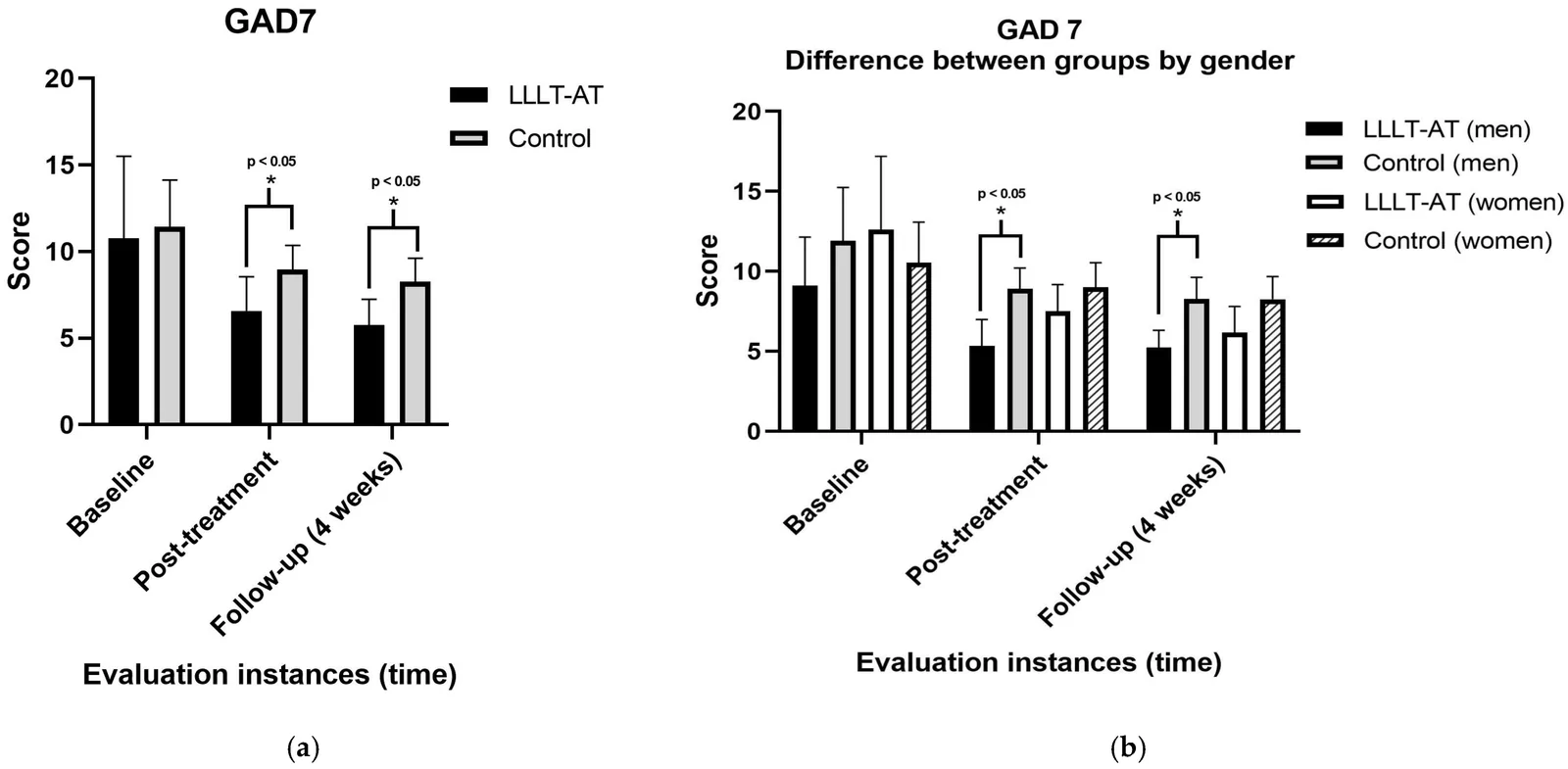
Comparison of anxiety levels (GAD-7) between the LLLT-AT and Sham LLLT-AT groups across the assessment time points. (**a**) Mean GAD-7 scores for both groups at baseline (T0), post-intervention (T1), and follow-up (T2); (**b**) Sex-stratified mean GAD-7 scores for the LLLT-AT and Sham LLLT-AT groups across the same assessment time points. *: Statistically significant difference (*p* < 0.05). **Abbreviations:** CI (confidence interval); CG (control group); DF (degrees of freedom); DFn (numerator degrees of freedom); DFd (denominator degrees of freedom); EG (experimental group); (GAD-7 (Generalized Anxiety Disorder-7); JFLS-8 (Jaw Functional Limitations Scale-8); LLLT-AT (low-level laser therapy auriculotherapy); MAS (masseter muscle); MMOROM (maximum opening range of motion); PPT (pressure pain threshold); SD (standard deviation); TEM (temporalis muscle).

A significant group × time interaction was observed for GAD-7 scores, supporting a differential effect of LLLT-AT compared to sham over time. At post-intervention (T1), the LLLT-AT group showed a greater reduction in GAD-7 scores compared with the Sham LLLT-AT group (mean difference −2.6; 95% CI −4.6 to −0.2; *p* = 0.02). This between-group difference persisted at follow-up (T2), favoring the LLLT-AT group (mean difference −2.5; 95% CI −4.67 to −0.3; *p* = 0.02). These differences are illustrated in Figure 4.

Based on group means and pooled standard deviations, between-group effect sizes for GAD-7 were large at both post-intervention (Hedges g = −1.35; 95% CI −2.00 to −0.70) and follow-up (Hedges g = −1.68; 95% CI −2.37 to −1.00), indicating a substantial reduction in anxiety levels in favor of the LLLT-AT group. Effect sizes for GAD-7 were large according to field-specific benchmarks for temporomandibular research [52]. These findings highlight a selective effect of LLLT-AT on psycho-emotional outcomes, with no additional benefit on mechanical or functional parameters.

### 3.5. Adverse Events

During the study, a single adverse event was reported. One participant in the LLLT-AT group experienced a mild headache following an assessment session, which led to voluntary withdrawal from the study before completion of the protocol. Given the timing of symptom onset, the event was considered potentially related to the algometric assessment procedure; however, it is also acknowledged that myogenic TMDs may be associated with headache symptoms, and therefore a direct causal relationship with the intervention could not be established [54].

No additional adverse events were reported in the LLLT-AT group or in the Sham LLLT-AT group during the intervention period or throughout the follow-up phase.

## 4. Discussion

This RCT examined the effects of LLLT-AT combined with a standardized myofascial release protocol in individuals with myogenic TMDs and comorbid anxiety. The main findings indicate that both the experimental and control interventions led to significant improvements in physical and functional outcomes. LLLT-AT, when used as an adjunct to manual therapy, was associated with greater reductions in anxiety compared with the sham intervention, with these differences persisting at follow-up. However, these findings should be interpreted with caution, given the reliance on a self-reported outcome and the potential influence of expectancy effects and response bias. Therefore, the results should not be interpreted as evidence of a specific biological mechanism, but rather as indicative of a potential adjunctive effect of LLLT-AT on psycho-emotional outcomes in this population.

### 4.1. Effects on Mandibular Outcomes

Improvements in PPT, MMOROM, and JFLS-8 were observed in both groups, with no statistically significant between-group differences. This pattern suggests that the observed gains in pain sensitivity, mandibular mobility, and functional performance were primarily driven by the standardized myofascial release protocol, which was applied equally in both groups [55]. Similar improvements in PPT and mandibular mobility following myofascial and pressure-based manual techniques have been reported in patients with myogenic TMDs [56,57,58]. From a mechanical and neuromuscular perspective, myofascial release may reduce masticatory muscle hypertonicity, improve tissue extensibility, and normalize mandibular movement patterns, thereby contributing to increased pressure tolerance and improved functional outcomes [55,57,58].

Auriculotherapy-based interventions, including LLLT-AT, have been proposed to influence pain processing through central and autonomic mechanisms, particularly via stimulation of auricular afferents connected to the autonomic nervous system. Experimental and neuroimaging evidence suggests that stimulation of the auricular branch of the vagus nerve may engage the nucleus tractus solitarius and downstream pain-regulatory networks, interacting with limbic and brainstem structures involved in the affective and motivational dimensions of pain [59,60]. In parallel, vagal activation has been associated with modulation of systemic inflammatory responses through the cholinergic anti-inflammatory pathway, providing a plausible neuroimmune mechanism for analgesic effects beyond purely peripheral modulation [61].

However, in the present trial, these hypothesized mechanisms did not translate into additional between-group improvements in PPT, MMOROM, or jaw functional limitation (JFLS-8) beyond those achieved with myofascial release alone. This finding may reflect that LLLT-AT did not provide additional benefit beyond an already effective standardized myofascial intervention for these somatic outcomes. Although a ceiling effect cannot be ruled out, this interpretation remains speculative, as post-intervention values were not formally compared with normative reference levels. Alternatively, it is possible that LLLT-AT did not provide an additional effect beyond the standardized myofascial intervention on these somatic outcomes. This pattern is consistent with previous evidence on LLLT-AT in musculoskeletal pain, where improvements in pain intensity are more consistently observed than changes in disability or pressure pain threshold, particularly when the certainty of evidence is low [28].

Additionally, baseline JFLS-8 values were relatively low, suggesting limited functional impairment in the study population. This may have reduced the potential to detect clinically meaningful between-group differences, as participants likely had restricted capacity for further functional improvement. Accordingly, the absence of additional effects of LLLT-AT on JFLS-8 outcomes should be interpreted considering the relatively low baseline levels of disability. Furthermore, mandibular functional limitation was included as a secondary outcome, and the study was not specifically powered to detect between-group differences in this variable.

Rather than diminishing the potential value of LLLT-AT, these results underscore the role of manual therapy as a foundational intervention for the musculoskeletal components of myogenic TMDs [55,58], while suggesting that the adjunctive effects of LLLT-AT may preferentially emerge in non-mechanical domains, such as psycho-emotional regulation [22].

### 4.2. Differential Effects of LLLT-AT on Anxiety

In contrast to the physical outcomes, anxiety—assessed using the GAD-7 scale—was the only variable showing a significant and sustained between-group difference, favoring the LLLT-AT group at both post-intervention and follow-up. Although the magnitude of anxiety reduction exceeded the minimal clinically important difference (MCID) for the GAD-7, commonly estimated at approximately 3–4 points [34,47,62], this threshold was reached primarily within the LLLT-AT group, whereas the between-group differences did not reach this level and remained modest. This highlights the need to distinguish between statistical significance and clinical relevance, as statistically significant differences may not necessarily translate into meaningful clinical benefits. Given the established role of anxiety in pain amplification, central sensitization, and symptom persistence in TMDs, this finding represents a potentially relevant but modest adjunctive effect rather than a definitive therapeutic advantage [11,13].

The differential improvement in anxiety cannot be explained by the myofascial release protocol alone, as this intervention was applied equally in both groups. This supports the hypothesis that LLLT-AT may exert an effect on psycho-emotional regulation. From a neurophysiological perspective, auricular stimulation is anatomically and functionally connected to central nervous system structures involved in emotional regulation, including the brainstem, limbic system, and prefrontal cortex. Sensory afferents associated with the auricular branch of the vagus nerve project to the nucleus tractus solitarius, a key integrative hub linking autonomic regulation with higher-order emotional and cognitive networks, such as the amygdala, anterior cingulate cortex, and prefrontal regions, as demonstrated by neuroimaging and neurophysiological studies [58,59]. Modulation of these circuits may attenuate hyperresponsivity in fear- and stress-related pathways, thereby contributing to reductions in anxiety symptoms.

In parallel, vagally mediated regulation of autonomic balance may play a central role. Enhancing parasympathetic activity and reducing sympathetic overactivation—frequently observed in anxiety and chronic pain conditions—may promote emotional stabilization. Additionally, modulation of the hypothalamic–pituitary–adrenal (HPA) axis represents a plausible mechanism, potentially leading to reduced secretion of stress-related hormones and improved neuroendocrine homeostasis. At a molecular level, engagement of anti-inflammatory pathways, including the cholinergic anti-inflammatory pathway via α7 nicotinic acetylcholine receptors, as well as neurochemical modulation involving endogenous opioids and serotonergic systems, has been proposed to contribute to the anxiolytic effects of auricular-based interventions [61,63].

While these mechanisms are supported by experimental and translational evidence [58,59,60,61], they should be interpreted as plausible explanatory pathways rather than definitive causal processes within the present trial. Together, these complementary central, autonomic, and neurochemical mechanisms provide a biologically plausible framework for the observed reduction in anxiety; however, these interpretations should be considered exploratory and not as confirmation of specific neurobiological mechanisms within the present trial. This interpretation is consistent with previous clinical findings showing improvements in anxiety-related outcomes following photobiomodulation auriculotherapy without parallel changes in mechanical or functional measures in TMDs and other clinical populations [26,64]. However, as anxiety was assessed using a self-reported measure and no objective autonomic or neuroendocrine markers were included, the possibility of expectancy effects and response bias cannot be excluded [65], and the absence of direct physiological measurements further limits mechanistic interpretation.

### 4.3. Sex-Related Considerations

An additional exploratory subgroup analysis by sex was conducted to descriptively examine potential differences in treatment response; however, these analyses were not pre-specified, and the study was not powered to detect subgroup effects. Exploratory analyses suggested that the anxiolytic effect of LLLT-AT was more pronounced in male participants, whereas women showed non-significant trends toward improvement. These findings should be interpreted in light of these considerations, as the small sample size within each sex group increases the likelihood that observed differences may reflect random variation rather than true biological effects.

Although sex-related differences in neuroendocrine and autonomic regulation have been described in the literature [66], their relevance to the present findings remains speculative and cannot be confirmed within the context of this study.

Taken together, these observations highlight the importance of considering sex as a relevant biological variable in future trials. Studies specifically designed and adequately powered to examine sex-related responses to auricular-based LLLT are warranted to clarify whether tailored intervention strategies may optimize therapeutic outcomes in individuals with myogenic TMDs and anxiety.

### 4.4. Complementary Role of LLLT-AT Within Physical Therapy

An integrative interpretation of the present findings is that myofascial release and LLLT-AT act on complementary levels of the pain–anxiety system. Manual therapy likely reduced peripheral nociceptive input and masticatory muscle hypertonicity, thereby decreasing somatic threat signals to the central nervous system and improving mechanical and functional outcomes. Within this therapeutic context, LLLT-AT may have preferentially targeted central and autonomic mechanisms, facilitating psycho-emotional regulation once peripheral contributors to pain were addressed, in line with multidimensional models of pain processing [61,64].

Within this biopsychosocial framework, the present findings suggest that LLLT-AT, when used as an adjunct to manual therapy, may preferentially modulate psycho-emotional dimensions, particularly anxiety [63,64,66]. Rather than supporting immediate widespread clinical implementation, these results point to a potential complementary neuromodulatory role of LLLT-AT and highlight the need for further mechanistic and clinically oriented research to better define its underlying mechanisms and clinical applicability.

From the perspective of new physical therapy insights, LLLT-AT offers several practical advantages. It is a non-invasive and generally well-tolerated intervention; however, its clinical implementation requires adherence to applicable legal regulations and safety standards for class IIIb laser devices, as well as appropriate professional training and competency in photobiomodulation. In addition, careful standardization of treatment parameters—including wavelength, dose, and application protocol—is essential to ensure safe and reproducible clinical outcomes. Its favorable safety profile and feasibility support its potential use in outpatient and community-based rehabilitation settings. Importantly, LLLT-AT directly addresses psycho-emotional factors—such as anxiety—that are frequently under-targeted in conventional musculoskeletal rehabilitation, despite their well-documented influence on pain perception, central sensitization, treatment responsiveness, and long-term outcomes [5,8,67,68]. In this context, emerging evidence suggests that LLLT-AT may exert clinically meaningful effects on pain and psycho-emotional outcomes while showing more variable or modest effects on mechanical and functional measures, particularly when used as an adjunct to standard physical therapy interventions [26,28,64,69].

In this sense, LLLT-AT should not be viewed as a substitute for established physical therapy interventions but rather as a complementary neuromodulatory strategy within a biopsychosocial framework. By combining peripheral mechanical interventions with auricular-based photobiomodulation, physical therapists may enhance the multidimensional management of myogenic TMDs, particularly in individuals presenting with clinically relevant anxiety. These findings support a stratified approach to physical therapy, in which adjunctive neuromodulatory strategies such as LLLT-AT may be particularly beneficial in patients with significant psycho-emotional involvement [28,70].

Furthermore, the present findings are based on a short-term follow-up period of four weeks, which does not allow conclusions regarding the durability or long-term clinical relevance of LLLT-AT in the management of chronic TMDs. Future studies with extended follow-up periods are required to determine the persistence and clinical significance of these effects over time.

### 4.5. Limitations and Future Directions

Several limitations should be acknowledged. Although the sample size was a priori calculated and adequate to detect the primary outcomes, it remains relatively limited for subgroup and exploratory analyses, such as sex-related comparisons. The sample size estimation was based on an assumed large effect size (approximately one standard deviation) derived from previous LLLT studies in masticatory muscle pain, particularly using PPT outcomes, which may not be fully transferable to auriculotherapy interventions; accordingly, the study should be interpreted as primarily powered to detect large between-group effects. Future studies should adopt more conservative and evidence-based assumptions for sample size estimation, in line with recent methodological recommendations in dentistry [69].

The follow-up period was restricted to four weeks, precluding conclusions regarding the long-term sustainability of the observed effects.

In addition, the study was conducted at a single center and involved a relatively young and homogeneous population, primarily composed of university students and staff, which may limit the generalizability of the findings to broader clinical populations. Furthermore, baseline stress levels and other psychosocial factors were not systematically evaluated and may represent relevant contributors to variability in anxiety outcomes.

Additionally, the therapist delivering the intervention was not blinded due to the nature of the treatment, which may introduce a potential risk of performance bias; however, participants and outcome assessors were blinded, and standardized procedures were implemented to minimize its impact. Although efforts were made to preserve participant blinding, complete blinding cannot be fully guaranteed in trials involving physical interventions. Therefore, the potential influence of expectancy effects on self-reported outcomes, such as anxiety, cannot be entirely excluded.

Furthermore, no direct measures of autonomic, inflammatory, or neuroendocrine activity were included, which limits the ability to confirm the proposed mechanisms underlying the anxiolytic effects of LLLT-AT. Therefore, the neurophysiological pathways discussed should be interpreted as plausible but inferential rather than as confirmed biological mechanisms.

Future studies should include larger samples to allow adequately powered subgroup analyses, extended follow-up periods, and objective measures of autonomic and stress-related regulation—such as heart rate variability, cortisol, or other neuroendocrine biomarkers—to further elucidate the mechanisms and durability of LLLT-AT effects. Future studies may also benefit from incorporating intermediate assessment time points during the intervention period to better characterize the temporal trajectory and onset of treatment effects. Incorporating additional psychosocial and central pain-related outcomes may also help to clarify the interaction between anxiety modulation and broader clinical responses. Identifying patient subgroups most likely to benefit from this intervention also represents an important direction for future research.

## 5. Conclusions

In individuals with myogenic TMDs, LLLT-AT combined with manual therapy did not demonstrate additional benefits over manual therapy alone for mechanical or functional outcomes, including PPT, MMROM, and functional limitation. However, adjunctive LLLT-AT was associated with greater reductions in anxiety compared with sham treatment, with effects maintained at short-term follow-up.

These findings suggest that auriculotherapy-based photobiomodulation may preferentially influence psycho-emotional dimensions rather than mechanical aspects of myogenic TMDs. This randomized controlled trial was prospectively designed with an a priori sample size calculation, and the relatively short follow-up period suggests that cautious interpretation is appropriate. Further randomized controlled trials with larger samples and longer follow-up are required to confirm these effects and to elucidate the underlying mechanisms.

## Figures and Tables

**Table 1:** Equipment Characteristics and Treatment Parameters for LLLT-AT.

Category	Parameter	Parameter Value
Equipment Parameters	Device	Combi 400L laser device (GymnaUniphy NV, Bilzen, Belgium). (As-Ga-Al diode)
	Wavelength (nm)	905 nm
	Peak power (W)	13.5 W
	Pulse duration (ns)	155 ns
	Pulse frequency (Hz)	133–30,000 Hz
Treatment Parameters for LLLT-AT	Average power (mW)	71 mW
	Spot size (cm^2^)	0.8 cm^2^
	Pulse frequency (Hz)	30,000 Hz
	Irradiance (mW/cm^2^)	89 mW/cm^2^
	Treatment time (s)	56 s per point (total 224 s)
	Energy per point (J)	4 J (4 points, total energy 16 J)
	Fluence (J/cm^2^)	4.0 J/cm^2^
	Application technique	Point technique with the laser probe placed perpendicular to the skin
	Number of sessions	6 (2 sessions per week)

**Abbreviations:** cm^2^: Square centimeter; Hz: Hertz; J: Joule; J/cm^2^: Joules per square centimeter (fluence); LLLT-AT: Low-level laser auriculotherapy; mW: Milliwatt; mW/cm^2^: Milliwatts per square centimeter (irradiance); nm: Nanometer; ns: Nanosecond; s: Second; W: Watt.

**Table 2:** Baseline demographic and clinical characteristics of participants in the LLLT-AT and control groups.

Variable	LLLT-AT Group (*n* = 23)	Control Group (*n* = 24)	Differences Between Groups (*p*-Value)
Age (years) mean (SD)	23.8 (5.6) ^n^	23.8 (5.5) ^n^	*p* = 0.88 ^†^
Sex men (n, %) women (n, %)	11 (47.8%) 12 (52.2%)	11 (45.8%) 13 (54.2%)	*p* = 0.99 ^ϲ^
PPT right MAS (N) mean (SD)	18.6 (6.7) ^n^	18.4 (6.9) ^n^	*p* = 0.90 ^†^
PPT right TEM (N) mean (SD)	25.5 (11.3) ^n^	23.6 (7.4) ^n^	*p* = 0.50 ^†^
PPT left MAS (N) mean (SD)	18.2 (6.5) ^n^	17.4 (6.8) ^n^	*p* = 0.71 ^†^
PPT left TEM (N) mean (SD)	24.1 (11.3) ^n^	22.3 (8.7) ^n^	*p* = 0.71 ^†^
MMOROM (cm) mean (SD)	3.7 (0.9) ^n^	3.8 (0.9) ^n^	*p* = 0.78 ^†^
JFLS-8 (score) mean (SD)	13.4 (9.0) ^n^	14.7 (10.2) ^n^	*p* = 0.62 ^†^
GAD-7 (Score) mean (SD)	10.8 (4.7) ^n^	11.4 (2.7) ^n^	*p* = 0.37 ^†^

**Notes:** Data are presented as mean ± standard deviation (SD), unless otherwise indicated. Values are presented for the randomized sample. ^c^ Chi-squared test; ^n^ Normal distribution assessed using the Shapiro–Wilk test; ^†^ Independent samples *t*-test. **Abbreviations:** GAD-7, Generalized Anxiety Disorder–7; JFLS-8, Jaw Functional Limitation Scale–8; LLLT-AT, low-level laser therapy auriculotherapy; MAS, masseter muscle; MMOROM, maximum mouth opening range of motion; N, Newtons; PPT, pressure pain threshold; SD, standard deviation; TEM, temporalis muscle.

**Table 3:** Within-group changes in clinical outcomes in the LLLT-AT group.

Variable	Baseline (T0) Mean (SD)	Post-Treatment (T1) Mean (SD)	Follow-Up (T2) Mean (SD)	Difference Between T0–T2 F (DF) (DFn, DFd) ^a^ (*p*-Value)	Change T1–T0 ^t^ Mean (CI 95%) (*p*-Value)	Change T2–T0 ^t^ Mean (CI 95%) (*p*-Value)	Change T2–T1 ^t^ Mean (CI 95%) (*p*-Value)
PPT right MAS (N)	18.6 (6.7) ^n,m^	25.6 (8.2) ^n,m^	23.6 (6.8) ^n,m^	F = 13.4 (2) (1.8, 34.8) *p* < 0.01 *	6.8 (1.4 to 12.1) *p* < 0.01 *	4.7 (1.5 to 8.4) *p* < 0.01 *	−1.9 (−4.6 to 0.7) *p* = 0.17
PPT right TEM (N)	25.5 (11.3) ^n,m^	30.9 (9.7) ^n,m^	32.1 (9.3) ^n,m^	F = 6.86 (2) (1.8, 35.6) *p* < 0.01 *	5.4 (0.2 to 10.5) *p* = 0.04 *	6.7 (1.4 to 12.0) *p* = 0.01 *	1.2 (2.6 to 5.2) *p* = 0.69
PPT left MAS (N)	18.2 (6.5) ^n,m^	26.7 (8.3) ^n,m^	25.0 (6.7) ^n,m^	F = 20.9 (2) (1.8, 36.9) *p* < 0.01 *	8.3 (4.5 to 12.3) *p* < 0.01 *	6.7 (3.7 to 9.7) *p* < 0.01 *	−1.7 (−5.1 to 1.7) *p* = 0.44
PPT left TEM (N)	24.1 (11.3) ^n,m^	33.2 (11.6) ^n,m^	32.2 (10.2) ^n,m^	F = 12.2 (2) (1.9, 37.9) *p* < 0.01 *	8.8 (3.4 to 14.3) *p* < 0.01 *	7.8 (3.3 to 12.3) *p* < 0.01 *	−1.0 (−5.8 to 3.8) *p* = 0.86
MMOROM (cm)	3.7 (0.9) ^n,m^	4.1 (0.9) ^n,m^	4.4 (0.9) ^n,m^	F = 15.8 (2) (1.9, 37.2) *p* < 0.01 *	0.4 (0.1 to 0.6) *p* < 0.01 *	0.7 (0.3 to 1.0) *p* < 0.01 *	0.3 (0.0 to 0.6) *p* = 0.06
JFLS-8 (score)	13.4 (9.0) ^n,m^	5.9 (4.6) ^n,m^	4.4 (5.7) ^n,m^	F = 28.1 (2) (2, 29.1) *p* < 0.01 *	−7.2 (−10.8 to −3.6) *p* < 0.01 *	−8.7 (−12.3 to −5.1) *p* < 0.01 *	−1.4 (−3.4 to 0.4) *p* = 0.16
GAD-7 (score)	10.8 (4.7) ^n,m^	6.6 (2.0) ^n,m^	5.8 (1.4) ^n,m^	F = 37.5 (2) (1.1, 22.0) *p* < 0.01 *	−4.1 (−5.9 to −2.4) *p* < 0.01 *	−5.0 (−7.0 to −3.0) *p* < 0.01 *	−0.8 (−1.4 to −0.2) *p* < 0.01 *

^a^: Repeated-measures analysis of variance (ANOVA). ^m^: Sphericity assessed using Mauchly’s test. ^n^: Normal distribution assessed using the Shapiro–Wilk test. ^t^: Tukey multiple comparisons test. *: Statistically significant difference (*p* < 0.05). **Abbreviations:** CI (confidence interval); DF (degrees of freedom); DFn (numerator degrees of freedom); DFd (denominator degrees of freedom); GAD-7 (Generalized Anxiety Disorder-7); JFLS-8 (Jaw Functional Limitations Scale-8); LLLT-AT (low-level laser therapy auriculotherapy); MAS (masseter muscle); MMOROM (maximum opening range of motion); N (Newton); PPT (pressure pain threshold); SD (standard deviation); TEM (temporalis muscle).

**Table 4:** Within-Group Outcomes in the Sham LLLT-AT Group.

Variable	Baseline (T0) Mean (SD)	Post-Treatment (T1) Mean (SD)	Follow-Up (T2) Mean (SD)	Difference Between T0–T2 F (DF) (DFn, DFd) ^a^ (*p*-Value)	Change T1–T0 ^t^ Mean (CI 95%) (*p*-Value)	Change T2–T0 ^t^ Mean (CI 95%) (*p*-Value)	Change T2–T1 ^t^ Mean (CI 95%) (*p*-Value)
PPT right MAS (N)	18.4 (6.9) ^n,m^	23.3 (7.2) ^n,m^	21.7 (6.5) ^n,m^	F = 10.5 (2) (1.6, 34.9) *p* < 0.01 *	4.7 (1.9 to 7.4) *p* < 0.01 *	3.1 (0.0 to 6.2) *p* = 0.04 *	−1.6 (−3.5 to 0.3) *p* = 0.11
PPT right TEM (N)	23.6 (7.4) ^n,m^	28.9 (8.5) ^n,m^	30.0 (7.0) ^n,m^	F = 9.9 (2) (1.5, 33.2) *p* < 0.01 *	5.1 (0.9 to 9.3) *p* = 0.02 *	6.2 (1.9 to 10.4) *p* < 0.01 *	1.0 (−1.3 to 3.5) *p* = 0.50
PPT left MAS (N)	17.4 (6.8) ^n,m^	23.3 (7.1) ^n,m^	23.1 (6.5) ^n,m^	F = 15.2 (2) (1.4, 31.4) *p* < 0.01 *	5.8 (2.3 to 9.2) *p* < 0.01 *	5.5 (2.1 to 8.9) *p* < 0.01	−0.23 (−2.0 to 1.6) *p* = 0.94
PPT left TEM (N)	22.3 (8.7) ^n,m^	29.7 (10.2) ^n,m^	30.8 (9.6) ^n,m^	F = 13.4 (2) (1.5, 33.5) *p* < 0.01 *	7.3 (2.4 to 12.3) *p* < 0.01 *	8.4 (3.4 to 13.4) *p* < 0.01 *	1.0 (−1.8 to 4.0) *p* = 0.65
MMOROM (cm)	3.8 (0.9) ^n,m^	4.2 (0.9) ^n,m^	4.3 (1.0) ^n,m^	F = 7.1 (2) (1.8, 40.1) *p* < 0.01 *	0.34 (0.1 to 0.68) *p* = 0.04 *	0.40 (0.1 to 0.7) *p* < 0.01 *	0.1 (−0.2 to 0.3) *p* = 0.86
JFLS-8 (score)	14.7 (10.2) ^n,m^	9.8 (7.6) ^n,m^	8.1 (9.8) ^n,m^	F = 12.9 (2) (1.4, 31.3) *p* < 0.01 *	−4.6 (−7.6 to −1.6) *p* < 0.01 *	−6.3 (−10.5 to −2.2) *p* < 0.01 *	−1.7 (−4.1 to 0.7) *p* = 0.19
GAD-7 (score)	11.4 (2.7) ^n,m^	8.9 (1.3) ^n,m^	8.2 (1.4) ^n,m^	F = 39.5 (2) (1.4, 32.4) *p* < 0.01 *	−2.5 (−3.5 to −1.4) *p* < 0.01 *	−3.2 (−4.2 to −2.1) *p* < 0.01 *	−0.7 (−1.3 to 0.1) *p* = 0.02 *

Note: Values reflect complete-case data (LLLT-AT: n = 21; control: n = 23). ^a^: Repeated -measures analysis of variance (ANOVA). ^m^: Sphericity assessed using Mauchly’s test. ^n^: Normal distribution assessed using the Shapiro–Wilk test. ^t^: Tukey multiple comparisons test. *: Statistically significant difference (*p* < 0.05). **Abbreviations:** CI (confidence interval); DF (degrees of freedom); DFn (numerator degrees of freedom); DFd (denominator degrees of freedom); (GAD-7 (Generalized Anxiety Disorder-7); JFLS-8 (Jaw Functional Limitations Scale-8); LLLT-AT (low-level laser therapy auriculotherapy); MAS (masseter muscle); MMOROM (maximum opening range of motion); PPT (pressure pain threshold); SD (standard deviation); TEM (temporalis muscle).

**Table 5:** Between-group comparisons of clinical outcomes between the LLLT-AT and Sham LLLT-AT groups.

Variable	Baseline		Post-Treatment		Follow-Up		Difference Between Groups F (DF) (DFn, DFd); ^a^ (*p*-Value)	Difference Between Groups (EG—CG) at T0 ^t^ Mean Difference (95% CI); (*p*-Value)	Difference Between Groups (EG—CG) at T1 ^t^ Mean Difference (95% CI); (*p*-Value)	Difference Between Groups (EG—CG) at T2 ^t^ Mean Difference (95% CI); (*p*-Value)
	LLLT-AT	Control	LLLT-AT	Control	LLLT-AT	Control				
PPT right MAS (N) men women	18.6 (6.7) ^n,l^ 22.4 (5.6) 15.7 (6.2)	18.4 (6.9) ^n,l^ 22.0 (8.7) 15.4 (3.0)	25.6 (8.2) ^n,l^ 32.0 (7.4) 20.8 (4.9)	23.3 (7.2) ^n,l^ 27.2 (6.2) 19.7 (6.4)	23.6 (6.8) ^n,l^ 28.5 (4.4) 20.0 (5.9)	21.7 (6.5) ^n,l^ 24.6 (6.6) 19.0 (5.4)	F = 0.26 (2) (2, 126); *p* = 0.77 F = 0.59 (2) (2, 55); *p* = 0.08 F = 0.04 (2) (2, 68); *p* = 0.96	0.3 (−5.9 to 6.5); *p* = 0.99 0.5 (−8.1 to 9.1); *p* > 0.99 0.3 (−5.9 to 6.5); *p* > 0.99	0.3 (−3.8 to 8.4); *p* = 0.88 4.8 (−4.0 to 13.7); *p* = 0.59 1.1 (−5.4 to 7.6); *p* = 0.99	1.9 (−4.2 to 1.8); *p* = 0.89 3.9 (−4.9 to 12.8); *p* = 0.78 1.0 (−5.5 to 7.4); *p* = 0.99
PPT right TEM (N) men women	25.5 (11.3) ^n^ 32.8 (10.9) 19.8 (8.1)	23.6 (7.4) ^n,l^ 27.4 (6.5) 20.4 (6.6)	30.9 (9.7) ^n,l^ 39.2 (7.4) 24.6 (5.5)	28.9 (8.5) ^n,l^ 33.0 (8.9) 25.2 (6.5)	32.1 (9.3) ^n,l^ 39.7 (5.1) 26.4 (7.5)	30.0 (7.0) ^n,l^ 32.5 (6.9) 27.8 (6.6)	F = 0.01 (2) (2, 126); *p* = 0.99 F = 0.07 (2) (2, 55); *p* = 0.93 F = 0.02 (2) (2, 68); *p* = 0.98	1.6 (−6.1 to 9.4); *p* = 0.99 5.4 (−4.7 to 15.6); *p* = 0.62 0.6 (−7.3 to 8.5); *p* = 0.99	1.95 (−5.8 to 9.7); *p* = 0.99 6.2 (−4.2 to 16.6); *p* = 0.50 0.6 (−7.7 to 8.8); *p* > 0.99	2.1 (−5.7 to 9.9); *p* = 0.97 7.3 (−3.2 to 17.7); *p* = 0.33 1.3 (−6.9 to 9.5); *p* = 0.99
PPT left MAS (N) men women	18.2 (6.5) ^n^ 22.2 (5.1) 15.0 (5.9)	17.4 (6.8) ^n,l^ 21.1 (7.7) 14.4 (4.2)	26.7 (8.3) ^n,l^ 32.0 (6.6) 22.8 (7.3)	23.3 (7.1) ^n,l^ 27.3 (6.4) 19.7 (5.9)	25.0 (6.7) ^n,l^ 30.2 (3.3) 21.1 (5.9)	23.1 (6.5) ^n,l^ 26.1 (6.8) 20.3 (5.1)	F = 0.39 (2) (2, 126); *p* = 0.68 F = 0.46 (2) (2, 55); *p* = 0.63 F = 0.36 (2) (2 68); *p* = 0.70	0.8 (−5.3 to 6.9); *p* = 0.59 1.2 (−6.8 to 9.2); *p* = 0.99 0.7 (−6.0 to 7.3); *p* = 0.99	3.4 (−2.7 to 9.5); *p* = 0.60 4.6 (−3.6 to 12.9); *p* = 0.57 3.1 (−3.8 to 10.1); *p* = 0.77	1.9 (−4.8 to 8.0); *p* = 0.61 4.2 (−4.1 to 12.4); *p* = 0.67 0.8 (−6.2 to 7.7); *p* = 0.99
PPT left TEM (N) men women	24.1 (11.3) ^n^ 32.1 (9.3) 18.0 (8.7)	22.3 (8.7) ^n,l^ 26.5 (8.0) 18.7 (7.9)	33.2 (11.6) ^n,l^ 43.0 (9.4) 25.9 (6.9)	29.7 (10.2) ^n,l^ 36.0 (10.5) 24.0 (5.8)	32.2 (10.2) ^n,l^ 40.1 (8.2) 26.3 (7.2)	30.8 (9.6) ^n,l^ 32.5 (11.2) 26.5 (5.5)	F = 0.12 (2) (2, 126); *p* = 0.89 F = 0.07 (2) (2, 55); *p* = 0.93 F = 0.25 (2) (2, 68); *p* = 0.78	2.0 (−6.9 to 11.3); *p* = 0.98 5.6 (−6.7 to 17.9); *p* = 0.76 0.7 (−7.4 to 8.9); *p* = 0.99	3.5 (−5.5 to 12.5); *p* = 0.86 7.0 (−5.7 to 19.6); *p* = 0.59 2.0 (−6.6 to 10.5); *p* = 0.98	1.4 (−7.5 to 10.4); *p* = 0.88 4.7 (−8.0 to 17.4); *p* = 0.88 0.2 (−8.3 to 8.8); *p* > 0.99
MMOROM (cm) men women	3.7 (0.9) ^n^ 4.3 (0.8) 3.3 (0.8)	3.8 (0.9) ^n,l^ 4.4 (1.0) 3.5 (0.6)	4.1 (0.9) ^n,l^ 4.5 (0.9) 3.9 (0.8)	4.2 (0.9) ^n,l^ 4.8 (0.8) 3.6 (0.6)	4.4 (0.9) ^n,l^ 4.9 (0.5) 4.0 (0.9)	4.3 (1.0) ^n,l^ 4.9 (0.7) 3.6 (0.7)	F = 0.25 (2) (2, 126); *p* = 0.85 F = 0.23 (2) (2, 55); *p* = 0.62 F = 0.88 (2) (2, 68); *p* = 0.41	−0.1 (−0.86 to 0.74); *p* = 0.99 −0.1 (−1.1 to 1.0); *p* > 0.99 −0.1 (−0.9 to 0.7); *p* = 0.99	−0.1 (−0.85 to 0.7); *p* = 0.99 −0.3 (−1.4 to 0.8); *p* = 0.96 −0.3 (−1.1 to 0.6); *p* = 0.94	−0.1 (−0.90 to 0.7); *p* = 0.99 0.1 (−1.1 to 1.1); *p* > 0.99 −0.5 (−1.3 to 0.4); *p* = 0.65
JFLS-8 (score) men women	13.3 (9.0) ^n^ 11.7 (6.9) 14.7 (10.1)	13.2 (10.2) ^n,l^ 12.3 (9.3) 16.7 (7.7)	5.9 (4.6) ^n,l^ 4.3 (3.4) 4.9 (2.2)	9.8 (7.6) ^n,l^ 7.4 (4.7) 12.1 (9.2)	4.4 (5.7) ^n,l^ 2.6 (3.4) 3.5 (3.5)	8.1 (9.8) ^n,l^ 5.4 (2.8) 10.6 (11.2)	F = 0.40 (2) (2, 126); *p* = 0.66 F = 0.29 (2) (2, 55); *p* = 0.15 F = 0.83 (2) (2, 68); *p* = 0.44	1.2 (−5.3 to 7.8); *p* = 0.99 −0.6 (−7.9 to 6.8); *p* = 0.99 −2.0 (−11.4 to 7.4); *p* = 0.99	−3.9 (−6.5 to 2.6); *p* = 0.52 −3.0 (−10.6 to 4.5); *p* = 0.84 −7.2 (−16.9 to 2.6); *p* = 0.27	−3.7 (−10.3 to 2.9); *p* = 0.90 −2.8 (−10.4 to 4.7); *p* = 0.88 −7.1 (−16.8 to 2.7); *p* = 0.28
GAD-7 (score) men women	10.5 (4.7) ^n^ 8.6 (3.2) 12.6 (4.6)	11.4 (2.7) ^n,l^ 11.9 (3.3) 10.5 (2.5)	6.6 (2.0) ^n,l^ 5.2 (1.7) 7.5 (1.7)	8.9 (1.3) ^n,l^ 8.9 (1.3) 9.0 (1.5)	5.8 (1.4) ^n,l^ 5.2 (1.1) 6.2 (1.6)	8.2 (1.4) ^n,l^ 8.3 (1.4) 8.3 (1.4)	F = 1.8 (2) (2, 126); *p* < 0.01 * F = 0.7 (2) (2, 55); *p* < 0.01 * F = 5.0 (2) (2, 68); *p* = 0.40	−0.6 (−2.9 to 1.6); *p* = 0.95 −2.7 (−7.1 to −1.3); *p* = 0.06 2.1 (−0.8 to 5.0); *p* = 0.31	−2.6 (−4.6 to −0.2); *p* = 0.02 * −3.6 (−6.5 to −0.6); *p* < 0.01 * −1.5 (−4.5 to 1.5); *p* = 0.70	−2.5 (−4.67 to −0.3); *p* = 0.02 * −3.1 (−6.0 to −0.1); *p* < 0.05 * −2.1(−5.1 to 0.9); *p* = 0.35

^a^: Two-way analysis of variance (ANOVA); ^n^: Normal distribution assessed using the Shapiro–Wilk test. ^l^: Homogeneity of variances assessed using Levene’s test. ^t^: Tukey multiple comparisons test. * Statistically significant difference (*p* < 0.05).

## Data Availability

Data are available from the corresponding author upon reasonable request.

## Abbreviations

The following abbreviations are used in this manuscript:

AL	Anxiety Level
ATP	Adenosine Triphosphate
CONSORT	Consolidated Standards of Reporting Trials
DC/TMD	Diagnostic Criteria for Temporomandibular Disorders
ICC	Intraclass Correlation Coefficient
LLLT-AT	Low-Level Laser Therapy–Auriculotherapy
MFL	Mandibular Functional Limitation
MCID	Minimal Clinically Important Difference
MMOROM	Maximum Mouth Opening Range of Motion
NSAIDs	Nonsteroidal Anti-Inflammatory Drugs
PENS	Percutaneous Electrical Nerve Stimulation
PPT	Pressure Pain Threshold
RCT	Randomized Controlled Trial
TENS	Transcutaneous Electrical Nerve Stimulation
TMDs	Temporomandibular Disorders
TMJ	Temporomandibular Joint
VAS	Visual Analogue Scale
